# Shedding and genetic diversity of *Coxiella burnetii* in Polish dairy cattle

**DOI:** 10.1371/journal.pone.0210244

**Published:** 2019-01-10

**Authors:** Monika Szymańska-Czerwińska, Agnieszka Jodełko, Kinga Zaręba-Marchewka, Krzysztof Niemczuk

**Affiliations:** 1 Department of Cattle and Sheep Diseases, National Veterinary Research Institute, Puławy, Poland; 2 Laboratory of Serological Diagnosis, National Veterinary Research Institute, Puławy, Poland; INRA, FRANCE

## Abstract

Q fever is a worldwide zoonotic disease reported in humans and many animal species including cattle. The aims of this study were to evaluate the prevalence of *Coxiella* (*C*.) *burnetii* shedding in Polish dairy cattle herds and to identify the pathogen’s genotypes and sequence types (STs) using multiple-locus variable number tandem repeat analysis (MLVA) and multispacer sequence typing (MST) methods. The presence of *C*. *burnetii* DNA was detected using a commercial real-time PCR kit, targeting the IS*1111* element. Overall, 1,439 samples from 279 herds were tested including: 897 individual milk specimens, 101 bulk tank milk samples, 409 genital tract swabs and 32 placentas. Furthermore, 30 consumer milk samples, including 10 from vending machines and 77 dairy products were also analyzed. *C*. *burnetii* shedding was confirmed in 31.54% of tested cattle herds as well as in 69.16% of consumer milk and dairy products. Among real-time PCR–positive samples, 49 specimens obtained from 49 cattle herds and 8 samples of purchased dairy products were selected for genotyping. Overall, five previously known MLVA genotypes (I, J, BG, BE, and NM) and three new ones (proposed as PL1, PL2, and PL3) were identified. Two MST sequence types were recorded: ST16 and a novel sequence (ST61). The new genotypes and sequence types need further research particularly into their pathogenicity to humans.

## Introduction

Q fever is a worldwide zoonotic infectious disease caused by *Coxiella* (*C*.) *burnetii–*an obligate intracellular bacterium. The range of this pathogen’s hosts is broad including domestic and wild mammals, birds and arthropods [1–3]. However, the domestic ruminants, mainly sheep and goats, are consider to be major reservoirs and source of infection for humans [4–6]. In these species, especially in cattle, *C*. *burnetii* infections are often asymptomatic. Ruminants may develop chronic disease, and syndromes including abortion, delivery of premature offspring, stillbirth and weak offspring (the APSW complex) may be observed [7]. APSW symptoms are rare in cattle, therefore even chronic infection may be imperceptible and confirmed only through laboratory tests.

In recent years, the number of reports from different countries about shedding of *C*. *burnetii* in cattle has been increasing [8]. Moreover, the Q fever epidemic in the Netherlands has contributed to intensification of the studies into molecular characterization of this pathogen in many countries. Also in Poland a serological monitoring programme for cattle and small ruminants has been implemented [9] since 2010. According to recently published data, herd-level seroprevalence in Polish cattle was estimated at 40.41%, which corresponds with European averages [10]. However, there is lack of information about current prevalence of *C*. *burnetii* in the cattle population in Poland. Moreover, data about genotypes circulating in these animals are very limited [11].

According to the last EFSA report, the numbers of confirmed Q fever cases in humans trended upwards over the period 2012–2016 in the EU [12]. Alsothere are still countries, including Poland, where human cases are not reported or are only sporadically. The epidemiological situation in animals taken in parallel with the small number of reported human cases may rather suggest that cases of Q fever in humans might have been underdiagnosed and underestimated. As is well known, infection is transmitted to humans predominantly by inhalation of contaminated aerosol droplets which can be spread by wind over long distances [13]. Infection by ingestion of raw milk or dairy products manufactured from unpasteurized milk remains debatable, however some studies indicate that these commodities can be a source of infection and pose a threat to human health [14–17]. The popularity of raw milk consumption has been increasing, which raises the risk of transmission of zoonotic agents to humans. Taking into consideration that asymptomatic cows shed *C*. *burnetii* predominantly in milk [18], monitoring surveys in dairy cattle herds are essential for protection of public health. Molecular characterization of *C*. *burnetii* is crucial for epidemiological investigation of Q fever outbreaks. Multispacer sequence typing (MST) and multiple-locus variable number tandem repeat analysis (MLVA) are recommended techniques which have been successfully utilized for genotyping by many researchers due to their high discriminatory power, reproducibility and possibility of application directly to DNA extracted from field samples without previous isolation of the pathogen [6, 19, 20]. Genotypic characterization of *C*. *burnetii* strains is vital to trace the source of the outbreak, to determine genotypes circulating in the population and to establish the potential connection between genotypes and virulence of the strains.

The aim of this study was to evaluate the prevalence of *C*. *burnetii* in Polish dairy cattle herds and also in consumer milk and dairy products in Poland, identify MST and MLVA genotypes, and compare these with those published in literature and databases.

## Materials and methods

### Samples and DNA extraction

**Fig 1:**
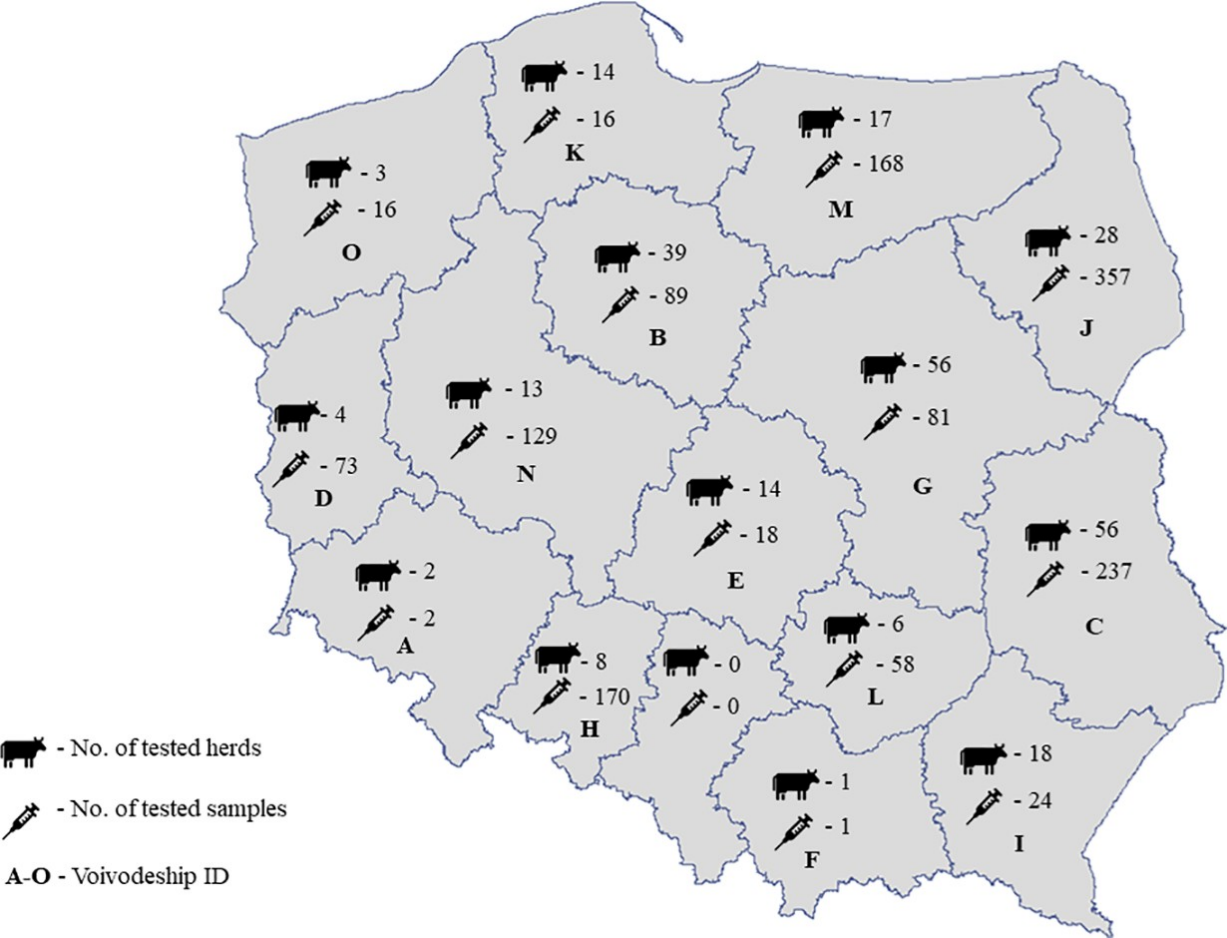
Number of herds and samples in Polish voivodeships tested by real-time PCR.

Sampling was performed between 2014 and 2017 in 15 out of 16 Polish voivodeships (Fig 1) excluding Silesia Province where samples were not available. Firstly, 279 dairy cattle herds were randomly selected in a cross-sectionally designed survey. Depending on availability, 30 ml individual milk samples, 100 ml bulk tank milk (BTM), sections of placenta containing at least three cotyledons, and vaginal swabs were collected. The latter were obtained from animals in postpartum period. The widest possible range of these four varieties of sample were collected from each herd. Detailed data about tested samples are present in S1 Table. Individual milk samples (n = 897), bulk tank milk (n = 101), vaginal swabs (n = 409), and placenta (n = 32) were subjected to a *C*. *burnetii-*specific real-time PCR targeting the IS*1111* repetitive element. The herd was classified as positive and underwent further research if at least one of the tested samples collected from the herd was positive in the real-time PCR.

Additionally, samples of consumer milk (n = 30), including milk from vending machines (n = 10), and dairy products (n = 77) manufactured by different companies were subjected to *C*. *burnetii*-specific qPCR. According to the information on the packaging all were produced by local dairies in Poland. Data presenting the types of tested dairy products and obtained results are summarized in S2 Table. If the isolation of nucleic acid was performed within 48h after a specimen was collected, the temperature of the sample was maintained between 4°C and 8°C, otherwise samples were stored at −20°C. DNA extraction from milk, placenta and swab samples was performed using a QIAamp DNA Mini Kit (Qiagen, Germany) according to the instructions of the manufacturer of the real-time PCR to be used next.

The process of nucleic acid extraction from dairy products was more complex and depended on their consistency. If the product’s density allowed precise pipetting, then 200 μl of the sample was taken. In other cases, products were homogenized and 25 mg of homogenate was taken for extraction. Then, 180 μl of ATL buffer and 20 μl of proteinase K were added to the sample prior to overnight incubation at 56 ±1°C. The follow-up procedure was performed as described in the QIAamp DNeasy Blood and Tissuse handbook (Qiagen, Germany). DNA aliquots were stored at –20°C until use.

### Ethics statement

According to the Local Ethical Committee on Animal Testing at the University of Life Sciences in Lublin (Poland), formal ethical approval is not required for this kind of study. Guidelines published by this ethics committee were consulted, which confirm that this work is sanctioned without specific ethical approval. All samples were taken by veterinarians during routine medical and veterinary activities, e.g. check-ups.

### Real-time PCR

All DNA extracts obtained both from samples collected from cattle (n = 1,439) and consumer bovine milk and dairy products (n = 107) were screened by an Adiavet COX Real Time commercial PCR kit (Adiagene, bioMérieux, France). The procedure was validated under laboratory conditions and accredited by the Polish Centre for Accreditation. A real-time PCR was performed using the 7500 Fast Real-Time PCR system v2.3 (Applied Biosystems, USA). A panel of required positive and negative controls was included in each run. An analytical cut-off value of 36.0 was selected corresponding to the defined lower limit of detection of the test.

### Genotyping (MLVA and MST)

Samples were selected for genotyping based on purity of DNA and cycle threshold (Ct) values obtained in the real-time PCR test. In total, 49 specimens from 49 herds and 8 samples of commercially available milk and dairy products were subjected to genotyping using MLVA and MST. Detailed information about selected specimens is presented in Table 1 (samples from cattle) and Table 2 (consumer milk and dairy products).

**Table 1:** Geographical location, number and type of samples selected for genotyping.

Voivodeship	No. of genotyped samples	Type of sample (herd ID)		
		Individual milk	BTM	Placenta
Kuyavia-Pomerania	10	B12, B23, B28, B39,	B1, B10, B11, B15, B36	B20
Lublin	8	C12, C17, C23, C24, C44	C16, C21, C55	-
Lubusz	1	-	D3	-
Łódź	2	E13	E12	-
Masovia	4	G14, G20, G22	G13	-
Opole	4	H3, H6, H8	H7	-
Podlasie	7	J2, J4, J11, J20, J22	J3, J28	-
Świętokrzyskie	1	L5	-	-
Warmia-Masuria	8	M1, M8, M14, M15	M4, M11, M16, M17	-
Greater Poland	2	N3, N10	-	-
West Pomerania	2	O1	O2	-

**Table 2:** Samples of milk and dairy products selected for genotyping.

Dairy product	Manufacturer’s ID	Ct values real-time PCR
raw milk	ML1	30.42
hard-ripened cheese	PR5	31.18
pasteurised milk	PR7	32.00
yogurt	PR9	32.58
cream cheese	PR19	29.43
smoked cheese	PR21	30.58
hard-ripened cheese	PR26	30.81
camembert cheese	PR31	31.02

### MLVA

MLVA was performed for the panel of 6 out of 17 loci published previously by Arricau-Bouvery et al. [21]. Two groups of markers were identified using primers published by Tilburg et al. [4] and Klaassen et al. [22] (Table 3): 3 heptanucleotide repeat markers (Ms23, Ms24 and Ms33) and 3 hexanucleotide repeat markers (Ms27, Ms28 and Ms34). PCR conditions were described elsewhere [11]. DNA of the Nine Mile RSA 493 strain, kindly provided by the Wageningen Bioveterinary Research (Lelystad, The Netherlands), was used as a reference and included in each run with all primer sets. The DNA amplification was carried out in a T-Personal 48 Thermocycler (Biometra, Germany). PCR products (0.5 μl) were diluted and mixed with 9 μl of Hi-Di formamide (Applied Biosystems, USA) and 0.5 μl of GeneScan 600 LIZ dye Size Standard (Applied Biosystems, USA). After denaturation for 3 minutes at 95°C, samples were cooled on ice. Analysis of the amplification products was performed on an ABI 3500 Genetic Analyser and electropherograms were evaluated with GeneMapper software v4.1 (Applied Biosystems, USA). The number of repeats in each locus was determined by extrapolating the sizes of the obtained fragments with those obtained for Nine Mile RSA 493 in the same run. Based on *in silico* analysis, the established genotype of this strain contained 9-27-4-6-9-5 repeats for marker loci Ms23-Ms24-Ms-27-Ms28-Ms33-Ms34, respectively. The MLVA genotype was identified as novel if the combination of repeats in all tested loci had not been described in the database [23] or research papers utilizing the same MLVA-6 method.

**Table 3:** Loci and primers used for MLVA analysis.

Locus	Primer name	Nucleotide sequence (5′→ 3′)	Length of STR [bp]	Amplicon size of Nine Mile strain [bp]	No. of STRs for Nine Mile strain	Reference
Ms23	Ms23F	FAM-CGCMTAGCGACACAACCAC	7	133	9	Tilburg et al. [4]
	Ms23R	GACGGGCTAAATTACACCTGCT				
Ms24	Ms24F	FAM-TGGAGGGACTCCGATTAAAA	7	261	27	Tilburg et al. [4]
	Ms24R	GCCACACAACTCTGTTTTCAG				
Ms27	Ms27F	FAM-TCTTTATTTCAGGCCGGAGT	6	89	4	Klaassen et al. [22]
	Ms27R	GAACGACTCATTGAACACACG				
Ms28	Ms28F	FAM-AGCAAAGAAATGTGAGGATCG	6	111	6	Klaassen et al. [22]
	Ms28R	GCCAAAGGGATATTTTTGTCCTTC				
Ms33	Ms33F	FAM-TCGCGTAGCGACACAACC	7	104	9	Tilburg et al. [4]
	Ms33R	GTAGCCCGTATGACGCGAAC				
Ms34	Ms34F	FAM-TTCTTCGGTGAGTTGCTGTG	6	101	5	Klaassen et al. [22]
	MS34R	GCAATGACTATCAGCGACTCGAA				

Clustering of obtained MLVA profiles was performed with Bionumerics v.7.6 software (Applied Maths, USA). Minimum spanning trees were generated to show the relationships between MLVA genotypes obtained in this study, the genotypes from the database [23] and those described by González-Barrio et al. [2], Piñero et al. [8] and Ceglie et al. [24]. In total, 459 samples (from cattle n = 202, humans n = 139, goats n = 53, sheep n = 36, ticks n = 11, European rabbits n = 10, red deer n = 6, rodents n = 1 and antelope n = 1) including six sequenced reference strains (Nine Mile RSA 493, Dugway 5J108-111, CbuG_Q212, CbuK_Q154, Cb175_Guyana, and Henzerling RSA 331) were used for comparison. A dendrogram was also constructed using the unweighted pair group method with arithmetic mean (UPGMA) to illustrate the genetic relationships between the MLVA profiles identified in this study and those described previously in Poland by Chmielewski et al. [11]. Only full MLVA genotypes were included in the analyses.

To assess the discriminatory power of the MLVA method for samples analyzed in this study, Hunter–Gaston diversity indices (HGDI) and corresponding 95% confidence intervals were calculated for each tested locus and for the overall MLVA method using the online tools http://www.hpa-bioinfotools.org.uk/cgi-bin/DICI/DICI.pl [25] and http://insilico.ehu.es/mini_tools/discriminatory_power/index.php [26], respectively. Only samples presenting complete MLVA profile were included in the calculation.

### MST

MST was performed as previously described by Glazunova et al. [19]. The ten different intergenic spacers Cox 2, 5, 18, 20, 22, 37, 51, 56, 57 and 61 were amplified and after purification the products were subjected to sequencing. Raw sequence data were assembled using Geneious Pro 8.0 software (Biomatters, New Zealand). Sequence types were determined based on the reference MST database available on the website [27].

## Results

### Real-time PCR

The overall herd-level prevalence of *C*. *burnetii* was calculated as 31.54% (88/279). The BTM samples gave positive real-time PCR results in 39.6% of herds, while individual milk specimens did so in 32.37% (Table 4). This percentage was lower for placenta (21.74%) and vaginal swab samples (11.54%). The real-time PCR test confirmed the presence of pathogen DNA in 165/897 individual milk samples, 40/101 of BTM, 166/407 vaginal swabs and 5/32 placentas.

**Table 4:** Results of real-time PCR.

Type of tested sample from one herd	No. of tested herds	No. of positive herds
individual milk	141	40
BTM	79	27
vaginal swab	7	0
placenta	14	2
individual and BTM	12	8
individual milk and vaginal swab	9	2
individual milk, vaginal swab and placenta	1	1
individual milk, BTM and vaginal swab	3	3
individual milk and placenta	6	1
individual milk, BTM,vaginal swab and placenta	1	1
BTM and placenta	1	1
BTM and vaginal swab	5	2
**Total**	**279**	**88**

### MLVA genotyping

**Fig 2:**
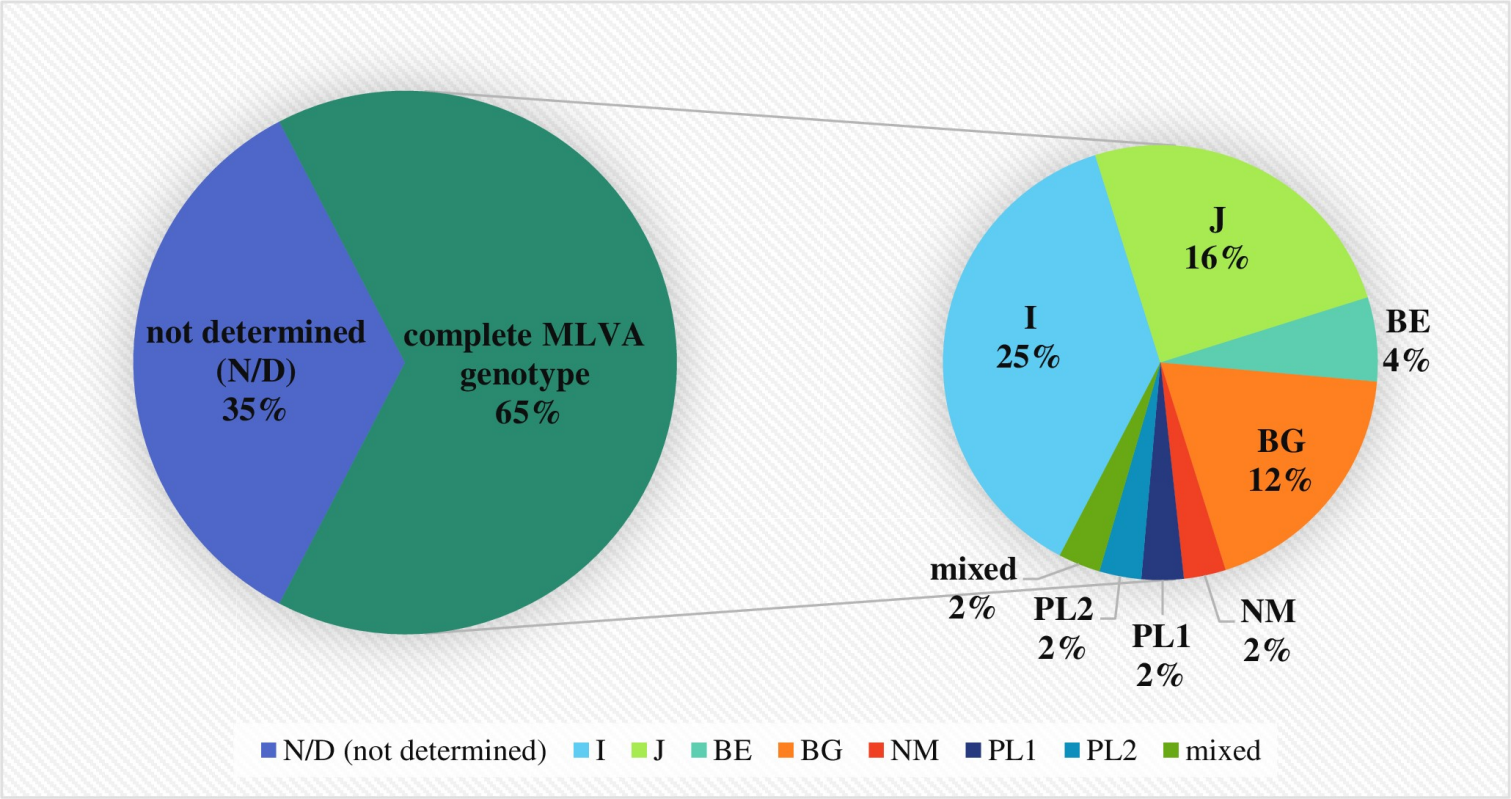
Frequency of MLVA genotype occurrence in tested cattle herds.

Detailed results of MLVA genotyping are presented in S3 Table. Seven complete MLVA genotypes were identified in 31 out of 49 samples originating from cattle herds and subjected to MLVA genotyping (Fig 2). The most common MLVA genotype was I (6-13-2-7-9-9), which was identified in 11 individual milk samples and one placenta specimen originating from six voivodeships. Genotype J (6-13-2-7-9-10) was found in six individual milk and two BTM samples from herds located in five voivodeships. The presence of genotype BG was confirmed in four individual milk and two BTM samples collected in 5 provinces. In one BTM sample from Lubusz Province and one individual milk specimen from Greater Poland Province, genotype BE (6-12-2-7-9-9) was identified. Genotype NM (9-27-4-6-9-5) was found in a BTM sample collected from a cattle herd located in Łódź Voivodeship.

Two of the MLVA genotypes identified in dairy cattle herds had not been described in online databases [23] or publications utilizing the same typing scheme. A genotype with the numbers of STRs 6-14-2-7-9-9 for loci Ms23-Ms24-Ms27-Ms28-Ms33-Ms34 (proposed name PL1) was present in a BTM sample originating from Lublin Province, while a second novel genotype (proposed name PL2) (6-13-2-7-9-7) was found in an individual milk sample collected in Greater Poland Voivodeship. In two analyzed BTM samples obtained from herds B15 and M11, two alleles in locus Ms34 were detected, which suggests the presence of mixed MLVA genotypes in the tested herds. Complete MLVA profiles were obtained in three samples of commercially available milk and dairy products for which the amplicons for all tested loci were obtained (S4 Table). Genotype I (6-13-2-7-9-9) was identified in a cream cheese sample and milk from a vending machine. In a camembert cheese sample, a third new MLVA genotype with the number of STRs 6-12-2-7-9-12 (proposed name PL3) was found. A mixed genotype was identified if more than one allele was present in the tested locus. This phenomenon was observed in two hard-ripened cheese samples in loci Ms24 and Ms34, and in smoked cheese sample only in locus Ms34.

**Fig 3:**
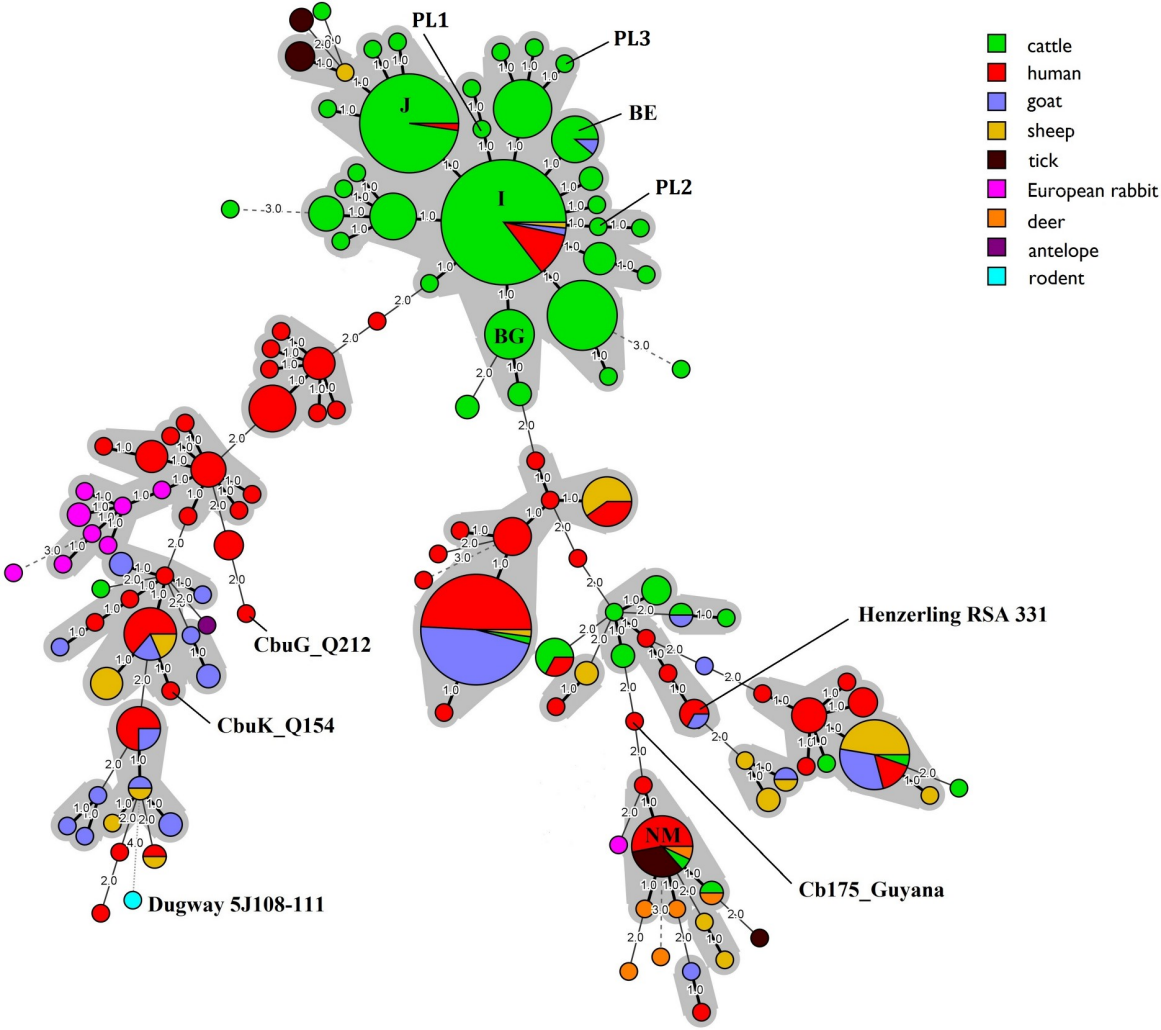
Minimum spanning tree showing the relationship between 459 MLVA genotypes identified in this study, collected in the database and reported elsewhere with reference to the host species. Only complete MLVA-6 profiles were included in the analysis.

**Fig 4:**
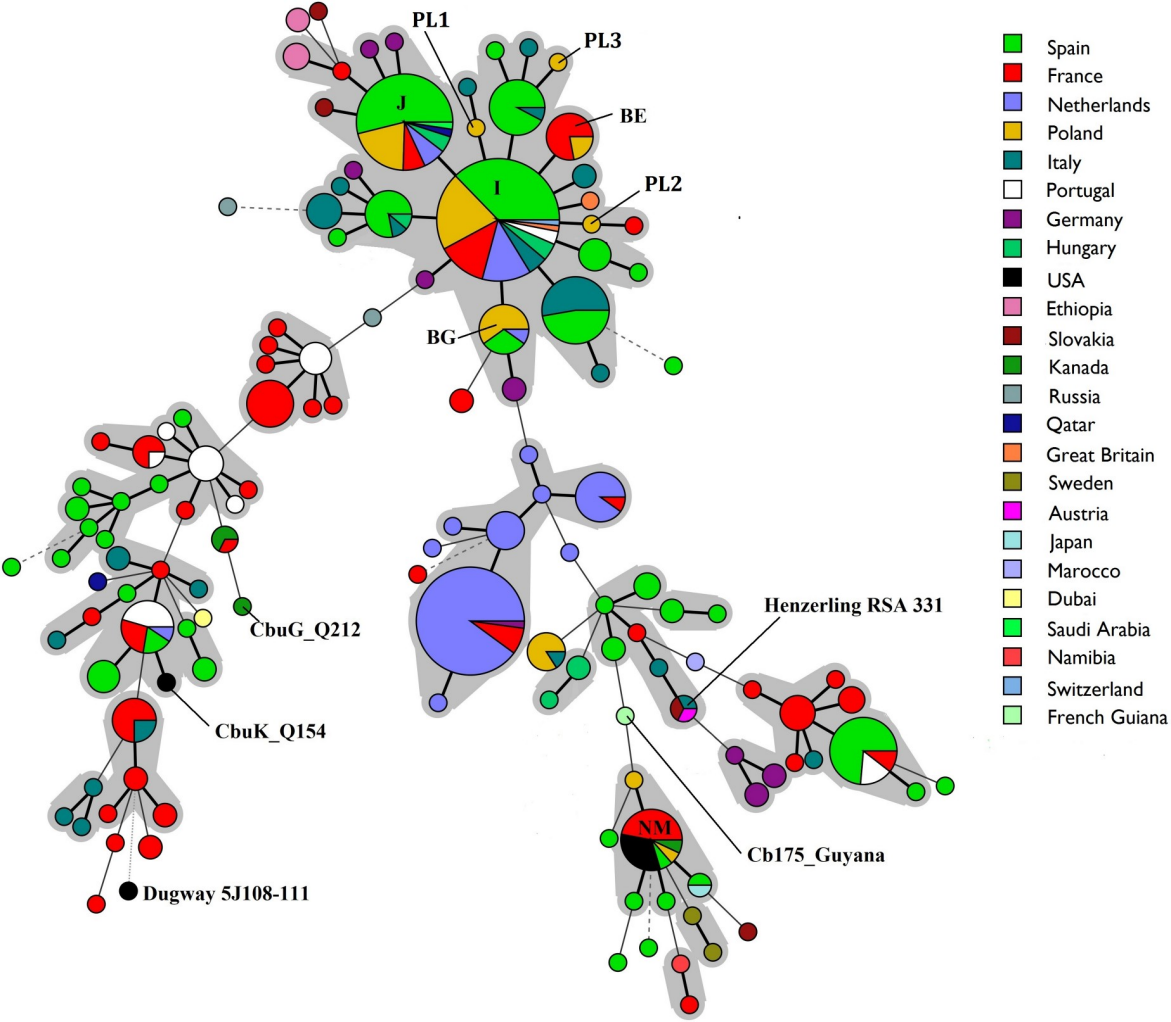
Minimum spanning tree showing the relationship between 459 MLVA genotypes identified in this study, collected in the database and reported elsewhere with reference to the geographical distribution. Only complete MLVA-6 profiles were included in the analysis.

Clustering of the MLVA genotypes using the minimum spanning tree method showed a high degree of genetic similarity between almost all of the identified genotypes. All but genotype NM were clustered together and interconnected by repeated number changes in one of the tested loci. Figs 3 and 4 present the relationship between all MLVA genotypes identified in this study, published in the previously described database and publications [2, 8, 24] regarding the animal host species and geographical distribution, respectively.

**Fig 5:**
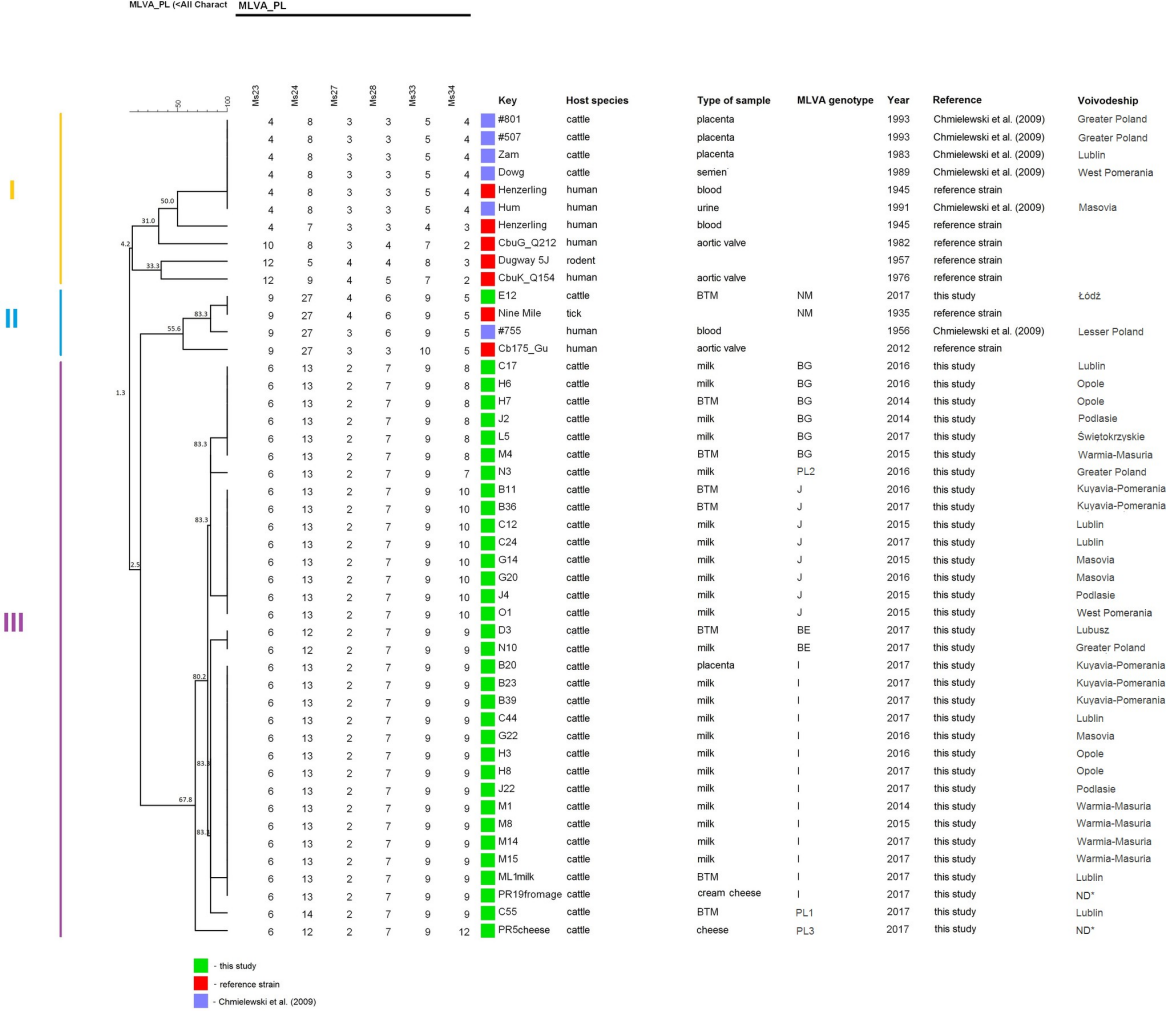
UPGMA cluster analysis of *C*. *burnetii* genotypes including 34 selected Polish samples from this study and from a study previously published by Chmielewski et al. [11] using multiple-locus variable-number tandem-repeat analysis. *not identified, lack of data about localization; blue squares–Chmielewski’s study [11]; red squares–reference strain; green squares–this study.

The UPGMA cluster analysis of the MLVA data revealed that genotypes of the strains analysed by Chmielewski et al. [11] and genotypes identified in this study are clearly separated in different clusters. Cluster I includes samples genotyped by Chmielewski [11], while almost all samples tested in this research (33/34) belong to cluster III and exhibit a high level of similarity. A sample from herd E12 is located separately in cluster II and shares 100% similarity with the genotype of Nine Mile RSA 493 (Fig 5).

The overall HGDI value of the MLVA method was estimated at 0.7594, calculated based on genotyping results of 34 samples for the panel of six tested loci. The HGDI value for a single locus ranged from 0 to 0.681. The highest value was noted for locus Ms34 (0.681), in which the presence of 6 alleles was observed, which indicates it was the most useful for genotyping. In contrast to locus Ms34, in locus Ms33 only one allele was present and in consequence this locus had no discriminatory power (Table 5).

**Table 5:** Hunter-Gaston diversity indices for each analysed MLVA locus.

Locus	No. of identified alleles	Range of repeats	HGDI	95% confidence interval
Ms23	2	6, 9	0.059	0.007–0.110
Ms24	4	12–14, 27	0.271	0.180–0.362
Ms27	2	2, 4	0.059	0.007–0.110
Ms28	2	6, 7	0.059	0.007–0.110
Ms33	1	9	0.0	0.000–0.184
Ms34	6	5, 7–10, 12	0.681	0.626–0.736

### MST genotyping

Detailed results of MST genotyping are presented in S5 Table. Alleles for all 10 tested loci were determined for 18 out of 49 genotyped samples using the MST method. Sequence analysis revealed that 17 of them represent a new sequence type. This sequence type differs from ST20 in locus Cox37, in which a deletion of a single nucleotide (T) at position 420 was noted compared to allele 4. After verification, the novel allele 37.10 for locus Cox37 was added to the online MST database (http://ifr48.timone.univ-mrs.fr/mst/coxiella_burnetii/) [27] and as a consequence the existence of the new sequence type named ST61 was confirmed. The allele profile of ST61 is 2-3-6-1-5-10-4-10-6-5 for intergenic spacers Cox2-Cox5-Cox18-Cox20-Cox22-Cox37-Cox51-Cox56-Cox57-Cox61, respectively. All but one of the tested samples belong to ST61, regardless of their MLVA genotypes. Analysis revealed that the bulk tank milk sample from herd E12, representing the NM genotype, belongs to ST16. Unfortunately, complete allelic profiles were not obtained for all samples. In two samples collected from herds C17 and M16 alleles in the spacer Cox37 were not identified, therefore they may belong to sequence type ST20 or ST61 (S5 Table).

For consumer milk and dairy products, a complete allelic profile was obtained only for one sample (cream cheese) and this specimen belonged to ST61. Moreover, in three samples (raw milk, hard-ripened cheese and smoked cheese) with partial MST profiles, the presence of allele 10 in locus Cox37 was detected, which suggests they may also represent ST61 (S6 Table).

## Discussion

The investigation performed in this study, revealed that *C*. *burnetii* is common in the cattle population in Poland which is consistent with the data from other countries [17]. The available literature show that herd-level prevalence of *C*. *burnetii* in cattle herds in European countries is differentiated, e.g. in northern Spain it is 52%, in Italy 40%, in Great Britain 29% and Portugal 20% [28–32]. In this research, shedding of the pathogen was confirmed by real-time PCR in almost 32% of tested bovine herds, which is congruent with average European percentages. *C*. *burnetii* DNA was detected in swabs from the reproductive tract and placentas. The most common was shedding in milk, which is in line with previous studies [18]. It should be taken into account that the percentage of infected herds may have been underestimated due to occurrence of intermittent shedding as well as the possibility of shedding by several routes [18, 33].

Genotyping of *C*. *burnetii* is essential for investigation of Q fever outbreaks as well as for analysis of the genomic heterogeneity of the pathogen. MLVA and MST methods were utilized in this study due to their high discriminatory power and possibility to be applied directly to DNA samples, without previous cultivation of the strain. Both methods revealed genotypic diversity among tested specimens, and in total eight MLVA genotypes and two sequence types of *C*. *burnetii* were identified. Five of these MLVA genotypes (I, J, BE, BG, NM) had been found before in other countries, mainly in samples collected from cattle. Analysis showed that genotypes I and J, which are widely distributed in this species worldwide, were also the most prevalent in this study. They were identified in almost 45% of genotyped samples in the study performed by Pinero et al. [8] in Spain. These genotypes were also found in milk and placentas from asymptomatic cattle in Hungary [34], while Astobiza et al. [35] found them in Spanish cattle herds with abortions and fertility disorders. Genotypes I and J were also dominant in the survey performed by Tilburg et al. [36], who tested 116 milk samples and dairy products available on the market in 28 countries. Genotype J was identified then in dairy products from Spain, Saudi Arabia, Qatar, Great Britain, France, and the Netherlands, while genotype I was revealed in France, Switzerland, the Netherlands, Spain and Portugal. In this study the presence of genotype I was recorded in an unpasteurized milk sample from a vending machine and in cream cheese sample. Genotype I was also identified in clinical samples e.g. heart valves, aorta tissue and placentas from humans in France in the 1990s and was also found incidentally in a goat in the Netherlands [37].

The third most prevalent genotype identified in this investigation was BG (6-13-2-7-9-8) which was detected in placenta collected from Dutch cattle and in bovine BTM in Spain [8]. Genotype BE (6-12-2-7-9-9) was recorded in a small percentage of tested herds. Previously its occurrence had been reported only in France, where it was found in bovine milk and placenta samples obtained from animals suffering from abortion and metritis [21] as well as in one goat milk sample. Taking into account the need for the protection of public health, it should be highlighted that the sample obtained from herd E12 belongs to the genotype NM and ST16 such as the Nine Mile RSA 493. This is significant because strains representing this genotype cause the acute form of Q fever in humans and have been previously recorded in ticks, blood samples and heart valves collected from humans in France and Canada [37]. Moreover, Gonzalez-Barrio et al. [2] confirmed the presence of this genotype in swabs from the reproductive tract of farmed red deer originating from a farm where cases of Q fever were noted both in animals and humans. Until the time of this study, genotype NM had not been recorded in cattle. It should be emphasized that three new MLVA genotypes were identified in this study. The first of them (PL1) with allelic profile 6-13-2-7-9-7 for loci Ms23-Ms24-Ms27-Ms28-Ms33-Ms34 differed from genotypes I, J and BG only in the number of short tandem repeats in locus Ms34. The second one (PL2) with allelic profile 6-14-2-7-9-9 had STR numbers in five loci identical to the numbers in genotypes I and BE, only locus Ms24 differing. The third genotype (PL3) identified in a sample of camembert cheese (6-12-2-7-9-12) differed from genotype BE in the number of STRs in locus Ms34 and from the other genotypes in loci Ms24 and Ms34. Analysis revealed that the majority of MLVA genotypes circulating in the cattle population in Poland show high genetic similarity and are interconnected by repeated number changes in one of the tested loci, except genotype NM whose allelic profile is different from the others in 5 out of 6 analyzed loci. Many researchers suggest that genotypes differing in the number of short tandem repeats might be microvariants of one genotype [24, 36]. Clustering of the MLVA genotypes using the minimum spanning tree method (Figs 3 and 4) showed high genetic similarity of genotypes identified in cattle from many countries, which may indicate a clonal spread of host-adapted *C*. *burnetii* strains in the cattle population all over the world [36]. Analysis of the frequency of occurrence of genotypes I, J, BG and BE in different regions of Poland revealed that their geographical distribution was wide and covered a few voivodeships often significantly far away from each other. More than one MLVA genotype was recorded in an area stretching over seven voivodeships. The highest diversity was noted in Lublin Province where four genotypes were recorded: I, J, BG and the new one, PL1. It is difficult to theorise about what the reason for the genetic diversity in this region may be.

Genotyping utilizing MST showed lower diversity of identified *C*. *burnetii* sequence types, which corresponds with results obtained by other researchers who point to the slightly lower discriminatory power of the MST method [37, 38]. The majority of samples obtained from cattle belong to the novel sequence type (ST61). The same ST was detected in the sample of cream cheese. Moreover, partial allelic profiles obtained for *C*. *burnetii* present in the three other dairy products indicate affinity with ST61. Galiero et al. [39] performed genotyping of *C*. *burnetii* DNA from dairy products but in this study they were manufactured from ovine and goat milk. The authors identified two STs: ST12 was identified in sheep cheese and mixed cheese sample, while ST32 was recorded in sheep cheese, and mixed cheese sample bovine milk. Both STs have a different allelic profile from ST61 in 8 out of 10 tested loci.

Detailed analysis of the new ST61 showed the highest similarity to ST20. The occurrence of ST20 in cattle is common and was noted in many countries. According to the investigations of Person et al. [40] and Bauer et al. [41], ST20 is currently the dominant sequence type recorded in bovine milk samples in USA. Despite its common prevalence in the world population of cattle, ST20 has very rarely been reported as a cause of infection for humans. Nevertheless, genotyping of *C*. *burnetti* strains isolated predominantly in France from humans suffering from chronic Q fever at the end of the 1990s showed that they belonged to ST20 [19]. Moreover genotype ST20 was identified in a Q fever outbreak associated with an abortion wave in a goat herd in Great Britain and also recorded in the same country in other outbreaks in farm animals but not in humans [42]. Due to the fact that the new sequence type ST61 has not been identified elsewhere in the world yet, there is no data about its virulence. The high resemblance of ST61 to ST20 may only indicate similar features of strains belonging to these two STs. It cannot be ruled out that differences in nucleotide sequences in the strains may have been localized also outside loci analyzed using MLVA and MST techniques. Therefore, further research using whole genome sequencing is indicated.

An important part of this research was evaluation of the variability of *C*. *burnetii* genotypes in Poland over time. Genotypes from this study were compared with genotypes of the source strains of Q fever outbreaks in cattle and humans in Poland in the 20^th^ century, including the strain which was responsible for the large outbreak recorded in 1982 in a south-eastern province of the country [11]. Analysis showed that currently tested samples represent different MLVA genotypes and sequence types than strains genotyped previously, with one exception. A sample collected from herd E12 was classified by the MST method as ST16 and to this sequence type also belonged the *C*. *burnetii* strain #755 isolated from the first outbreak of Q fever in Poland from a patient with an acute flu-like form of the disease [11]. According to available online databases, ST16 shows a wide geographical distribution spanning the four continents of Europe, Asia, Africa and North America. ST16 was detected in bovine milk samples from the USA and Japan as well as in placenta and milk from German cattle. Moreover, it was detected in blood samples and less frequently in heart valves sampled from humans with the acute form of Q fever in France, Italy, the USA, Central Africa and Romania [19].

## Conclusion

In conclusion, this research provides new data about the prevalence and genotypic diversity of *C*. *burnetii* in Polish dairy cattle herds and consumer dairy products. The investigation revealed that shedding of *C*. *burnetii* in dairy cattle herds in Poland is common, which was confirmed by the high percentage of positive milk and dairy products specimens. In total, eight MLVA genotypes including three new ones (PL1, PL2, PL3) and two sequence types including one new one (ST61) were identified. It should be highlighted that the MLVA and MST profiles identified in this study were different from profiles of the strain involved in the Q fever outbreak in the Netherlands as well as from genotypes of the outbreak strains isolated in Poland in the 20^th^ century. Moreover, some of them have been previously recorded in humans as genotypes I, J, NM and ST16, therefore a zoonotic threat cannot be ruled out. The pathogenicity to humans of new genotypes needs further research.

## Supporting information

S1 Table Detailed data about tested samples. (DOCX)

S2 Table Type of tested milk and dairy products and qPCR results. (DOCX)

S3 Table Results of MLVA genotyping. ND ‒ not determined, due to lack of product amplification for all tested loci
*according to nomenclature proposed by Tilburg [35]
(DOCX)

S4 Table Results of MLVA genotyping of milk and dairy products. ND ‒ not determined, due to lack of product amplification for all tested loci
*according to nomenclature proposed by Tilburg [35]
(DOCX)

S5 Table Results of genotyping using MST method. ND–not determined
*Sequence type established based on incomplete allelic profile
** according to online database [26]
(DOCX)

S6 Table Results of genotyping of milk and dairy products using MST method. ND–not determined
*Sequence type established based on incomplete allelic profile
** according to online database [26]
(DOCX)
