# Supplementary material for: Shedding and genetic diversity of Coxiella burnetii in Polish dairy cattle
Source: PLoS One. 2019 Jan 10;14(1):e0210244. doi: 10.1371/journal.pone.0210244 (PMC6328121; doi:10.1371/journal.pone.0210244)
Supplement: S1 Table — (DOCX) [file pone.0210244.s001.docx]

**S1 Table.** Detailed data about tested samples.

| **Voivodeship (ID)** | **No. of tested herds** | **Herd ID** | **No of tested animals*** | **No. of tested samples**  **(No. of positive samples)** | | | | **No. of positive animals** | **No. of positive herds (%)** |
| --- | --- | --- | --- | --- | --- | --- | --- | --- | --- |
|  |  |  |  | **Individual milk** | **BTM** | **Vaginal swab** | **Placenta** |  |  |
| **Lower-Silesia (A)** | 2 | A1 | ND | - | 1 (1) | - | - | ND | 1 (50) |
|  |  | A2 | ND | - | 1 (0) | - | - | 0 |  |
| **Kuyavia-Pomerania (B)** | 39 | B1 | ND | - | 1 (1) | - | - | ND | 15 (38.46) |
|  |  | B2 | 1 | 1 (0) | - | - | - | 0 |  |
|  |  | B3 | 1 | 1 (0) | - | - | - | 0 |  |
|  |  | B4 | ND | 1 (0) | 1 (0) | - | - | 0 |  |
|  |  | B5 | 1 | 1 (0) | - | - | - | 0 |  |
|  |  | B6 | 1 | 1 (0) | - | - | - | 0 |  |
|  |  | B7 | ND | - | 1 (1) | - | - | 1 |  |
|  |  | B8 | 1 | 1 (0) | - | - | - | 0 |  |
|  |  | B9 | 1 | 1 (0) | - | - | - | 0 |  |
|  |  | B10 | ND | - | 1 (1) | - | - | ND |  |
|  |  | B11 | ND | - | 1 (1) | - | - | ND |  |
|  |  | B12 | 1 | 1 (1) | - | - | - | 1 |  |
|  |  | B13 | 1 | 1 (0) | - | - | - | 0 |  |
|  |  | B14 | ND | - | 1 (1) | - | - | ND |  |
|  |  | B15 | ND | - | 1 (1) | - | - | ND |  |
|  |  | B16 | 1 | 1 (0) | - | - | - | 0 |  |
|  |  | B17 | 1 | 1 (0) | - | - | - | 0 |  |
|  |  | B18 | 1 | 1 (1) | - | - | - | 1 |  |
|  |  | B19 | 1 | 1 (0) | - | - | - | 0 |  |
|  |  | B20 | 14 | 14 (4) | - | - | 1 (1) | 4 |  |
|  |  | B21 | 1 | 1 (0) | - | - | - | 0 |  |
|  |  | B22 | 1 | 1 (0) | - | - | - | 0 |  |
|  |  | B23 | 1 | 1 (1) | - | - | - | 1 |  |
|  |  | B24 | ND | - | 1 (0) | - | - | 0 |  |
|  |  | B25 | 1 | 1 (0) | - | - | - | 0 |  |
|  |  | B26 | 1 | 1 (0) | - | - | - | 0 |  |
|  |  | B27 | ND | - | 1 (0) | - | - | 0 |  |
|  |  | B28 | 1 | 1 (1) | - | - | - | 1 |  |
|  |  | B29 | ND | - | 1 (1) | - | - | ND |  |
|  |  | B30 | 1 | 1 (0) | - | - | - | 0 |  |
|  |  | B31 | ND | - | 1 (0) | - | - | 0 |  |
|  |  | B32 | ND | - | 1 (0) | - | - | 0 |  |
|  |  | B33 | ND | - | 1 (1) | - | - | ND |  |
|  |  | B34 | ND | - | 1 (0) | - | - | 0 |  |
|  |  | B35 | ND | - | 1 (0) | - | - | 0 |  |
|  |  | B36 | ND | - | 1 (1) | - | - | ND |  |
|  |  | B37 | ND | - | 1 (0) | - | - | 0 |  |
|  |  | B38 | ND | - | 1 (0) | - | - | 0 |  |
|  |  | B39 | 36 | 36 (5) | - | - | - | 5 |  |
| **Lublin (C)** | 56 | C1 | ND | - | 1 (1) | - | - | ND | 17 (30.36) |
|  |  | C2 | 2 | 2 (2) | - | - | - | 2 |  |
|  |  | C3 | 1 | 1 (1) | - | - | - | 1 |  |
|  |  | C4 | 1 | 1 (0) | - | - | - | 0 |  |
|  |  | C5 | 1 | 1 (1) | - | - | - | 1 |  |
|  |  | C6 | 4 | 4 (2) | - | - | - | 2 |  |
|  |  | C7 | 1 | 1 (0) | - | - | - | 0 |  |
|  |  | C8 | 1 | 1 (0) | - | - | - | 0 |  |
|  |  | C9 | 1 | 1 (0) | - | 1 (0) | - | 0 |  |
|  |  | C10 | ND | - | 1(0) | - | - | 0 |  |
|  |  | C11 | ND | - | 1 (0) | - | - | 0 |  |
|  |  | C12 | 46 | 46 (9) | - | 10 (7) | 2 (1) | 16 |  |
|  |  | C13 | 1 | 1 (0) | - | - | - | 0 |  |
|  |  | C14 | 1 | 1 (1) | - | - | - | 1 |  |
|  |  | C15 | 1 | 1 (1) | - | - | - | 1 |  |
|  |  | C16 | ND |  | 1 (1) | - | - | ND |  |
|  |  | C17 | 1 | 1 (1) | - | - | - | 1 |  |
|  |  | C18 | 1 | 1 (0) | - | - | - | 0 |  |
|  |  | C19 | 1 | 1 (0) | - | - | - | 0 |  |
|  |  | C20 | ND | - | 1 (0) | - | - | 0 |  |
|  |  | C21 | ND | - | 1 (1) | - | - | ND |  |
|  |  | C22 | 1 | 1 (0) | - | - | - | 0 |  |
|  |  | C23 | 1 | 1 (1) | - | - | - | 1 |  |
|  |  | C24 | 1 | 1 (1) | - | - | - | 1 |  |
|  |  | C25 | 1 | 1 (1) |  |  |  | 1 |  |
|  |  | C26 | 1 | - | - | 1 (0) | - | 0 |  |
|  |  | C27 | 4 | - | - | 4 (0) | - | 0 |  |
|  |  | C28 | 2 | 2 (0) | - | - | 2 (0) | 0 |  |
|  |  | C29 | 1 | 1 (0) | - | - | 1 (0) | 0 |  |
|  |  | C30 | 1 | 1 (0) | - | - | 1 (0) | 0 |  |
|  |  | C31 | 10 | - | - | 10 (0) | - | 0 |  |
|  |  | C32 | 1 | 1 (0) | - | - | 1 (0) | 0 |  |
|  |  | C33 | 15 | 15 (0) | - | 15 (0) | - | 0 |  |
|  |  | C34 | ND | - | 1 (0) | 10 (0) | - | 0 |  |
|  |  | C35 | ND | - | 1 (0) | 6 (0) | - | 0 |  |
|  |  | C36 | ND | - | 1 (0) | 10 (0) | - | 0 |  |
|  |  | C37 | 1 | 1 (0) | - | - | - | 0 |  |
|  |  | C38 | 2 | - | - | - | 2 (1) | 1 |  |
|  |  | C39 | ND | - | 1 (0) | - | - | 0 |  |
|  |  | C40 | ND | 1 (0) | 1 (0) | - | - | 0 |  |
|  |  | C41 | 1 | 1 (0) | - | - | - | 0 |  |
|  |  | C42 | 5 | 5 (0) | - | - | - | 0 |  |
|  |  | C43 | ND | - | 1 (0) | - | - | 0 |  |
|  |  | C44 | 11 | 11 (2) | - | - | - | 2 |  |
|  |  | C45 | ND | - | 1 (0) | - | - | 0 |  |
|  |  | C46 | ND | - | 1 (0) | - | - | 0 |  |
|  |  | C47 | ND | - | 1 (0) | - | - | 0 |  |
|  |  | C48 | ND | - | 1 (0) | - | - | 0 |  |
|  |  | C49 | 1 | 1 (0) | - | - | - | 0 |  |
|  |  | C50 | 1 | 1 (0) | - | - | - | 0 |  |
|  |  | C51 | ND | - | 1 (0) | - | - | 0 |  |
|  |  | C52 | ND | - | 1 (0) | - | - | 0 |  |
|  |  | C53 | ND | - | 1 (0) | - | - | 0 |  |
|  |  | C54 | ND | - | 1 (0) | - | - | 0 |  |
|  |  | C55 | ND | 20 (1) | 1 (1) | 10 (0) | - | ND |  |
|  |  | C56 | ND | - | 1 (0) | - | - | 0 |  |
| **Lubusz (D)** | 4 | D1 | 42 | 29 (0) | - | 41 (0) | - | 0 | 2 (50) |
|  |  | D2 | 1 | 1 (1) | - | - | - | 1 |  |
|  |  | D3 | ND | - | 1 (1) | - | - | ND |  |
|  |  | D4 | 1 | 1 (0) | - | - | - | 0 |  |
| **Łódź (E)** | 14 | E1 | ND | 1 (0) | 1 (0) | - | - | 0 | 2(14.29) |
|  |  | E2 | ND | - | 1 (0) | - | - | 0 |  |
|  |  | E3 | ND | - | 1 (0) | - | - | 0 |  |
|  |  | E4 | 1 | 1 (0) | - | - | - | 0 |  |
|  |  | E5 | 1 | 1 (0) | - | - | - | 0 |  |
|  |  | E6 | 1 | 1 (0) | - | - | - | 0 |  |
|  |  | E7 | ND | - | 1 (0) | - | - | 0 |  |
|  |  | E8 | ND | - | 1 (0) | - | - | 0 |  |
|  |  | E9 | ND | - | 1 (0) | - | - | 0 |  |
|  |  | E10 | 1 | 1 (0) | - | - | - | 0 |  |
|  |  | E11 | ND | - | 1 (0) | - | - | 0 |  |
|  |  | E12 | ND | 1 (1) | 1 (1) | - | - | ND |  |
|  |  | E13 | ND | 2 (1) | 1 (0) | - | - | 1 |  |
|  |  | E14 | 1 | 1 (0) | - | - | - | 0 |  |
| **Lesser Poland (F)** | 1 | F1 | 1 | 1 (0) | - | - | - | 0 | 0 |
| **Masovia (G)** | 56 | G1 | ND | - | 1 (1) | - | - | ND | 9 (16.07) |
|  |  | G2 | ND | - | 1 (0) | - | - | 0 |  |
|  |  | G3 | ND | - | 1 (0) | - | - | 0 |  |
|  |  | G4 | ND | - | 1 (0) | - | - | 0 |  |
|  |  | G5 | ND | - | 1 (0) | - | - | 0 |  |
|  |  | G6 | ND | - | 1 (0) | - | - | 0 |  |
|  |  | G7 |  | - | 1 (0) | - | - | 0 |  |
|  |  | G8 | 1 | 1 (0) | - | - | - | 0 |  |
|  |  | G9 | 1 | 1 (0) | - | - | - | 0 |  |
|  |  | G10 | 2 | - | - | 2 (0) |  | 0 |  |
|  |  | G11 | 1 | - | - | - | 1 (0) | 0 |  |
|  |  | G12 | ND | - | 1 (1) | - | - | ND |  |
|  |  | G13 | ND | 2 (0) | 1 (1) | - | - | ND |  |
|  |  | G14 | 1 | 1 (1) | - | - | - | 1 |  |
|  |  | G15 | 2 | 2 (0) | - | - | - | 0 |  |
|  |  | G16 | 9 | 9 (0) | - | - | - | 0 |  |
|  |  | G17 | 1 | 1 (0) | - | - | - | 0 |  |
|  |  | G18 | 1 | 1 (0) | - | - | - | 0 |  |
|  |  | G19 | ND | - | 1 (0) | - | - | 0 |  |
|  |  | G20 | ND | 3 (1) | 1 (0) | - | - | 1 |  |
|  |  | G21 | ND | 2 (0) | - | - | - | 0 |  |
|  |  | G22 | 1 | 1 (1) | - | - | - | 1 |  |
|  |  | G23 | 1 | - | - | 1 (0) | - | 0 |  |
|  |  | G24 | 1 | - | - | - | 1 (0) | 0 |  |
|  |  | G25 | ND | - | 1 (0) | - | - | 0 |  |
|  |  | G26 | ND | - | 1 (0) | - | - | 0 |  |
|  |  | G27 | 2 | 2 (0) | - | - | - | 0 |  |
|  |  | G28 | 1 | 1 (0) | - | - | - | 0 |  |
|  |  | G29 | 1 | 1 (0) | - | - | - | 0 |  |
|  |  | G30 | 1 | 1 (1) | - | - | - | 1 |  |
|  |  | G31 | 1 | 1 (0) | - | - | - | 0 |  |
|  |  | G32 | 1 | 1 (0) | - | - | - | 0 |  |
|  |  | G33 | 1 | 1 (0) | - | - | - | 0 |  |
|  |  | G34 | 1 | 1 (0) | - | - | - | 0 |  |
|  |  | G35 | 1 | 1 (0) | - | - | - | 0 |  |
|  |  | G36 | 1 | 1 (0) | - | - | - | 0 |  |
|  |  | G37 | 1 | 1 (0) | - | - | - | 0 |  |
|  |  | G38 | 1 | 1 (0) | - | - | - | 0 |  |
|  |  | G39 | 1 | 1 (1) | - | - | - | 1 |  |
|  |  | G40 | 1 | 1 (0) | - | - | - | 0 |  |
|  |  | G41 | 1 | 1 (0) | - | - | - | 0 |  |
|  |  | G42 | 1 | 1 (0) | - | - | - | 0 |  |
|  |  | G43 | 1 | 1 (0) | - | - | - | 0 |  |
|  |  | G44 | 1 | 1 (0) | - | - | - | 0 |  |
|  |  | G45 | 1 | 1 (0) | - | - | - | 0 |  |
|  |  | G46 | 1 | 1 (0) | - | - | - | 0 |  |
|  |  | G47 | 1 | 1 (0) | - | - | - | 0 |  |
|  |  | G48 | 2 | - | - | - | 2 (1) | 1 |  |
|  |  | G49 | 1 | 1 (0) | - | - | - | 0 |  |
|  |  | G50 | 1 | 1 (0) | - | - | - | 0 |  |
|  |  | G51 | 1 | 1 (0) | - | - | - | 0 |  |
|  |  | G52 | 7 | 7 (0) | - | - | 1 (0) | 0 |  |
|  |  | G53 | ND | - | 1 (0) | - | - | 0 |  |
|  |  | G54 | 1 | 1 (0) | - | - | - | 0 |  |
|  |  | G55 | 1 | 1 (0) | - | - | - | 0 |  |
|  |  | G56 | ND | - | 1 (0) | - | - | 0 |  |
| **Opole (H)** | 8 | H1 | ND | 5 (3) | 1 (1) | - | - | ND | 5 (62.5) |
|  |  | H2 | 2 | 2 (0) | - | - | - | 0 |  |
|  |  | H3 | 3 | 3 (2) | - | - | - | 2 |  |
|  |  | H4 | 1 | 1 (0) | - | - | - | 0 |  |
|  |  | H5 | 1 | 1 (0) | - | - | - | 0 |  |
|  |  | H6 | 1 | 1 (1) | - | - | - | 1 |  |
|  |  | H7 | ND | - | 1 (1) | - | 2 (1) | ND |  |
|  |  | H8 | 152 | 152 (26) | 1 (1) | - | - | 26 |  |
| **Subcarpathia (I)** | 18 | I1 | 1 |  | - | - | 1 (0) | 0 | 3 (16.67) |
|  |  | I2 | 2 | 2 (0) | - | 2 (0) | - | 0 |  |
|  |  | I3 | 2 | 2 (0) |  | 2 (0) |  | 0 |  |
|  |  | I4 | 1 | 1 (0) | - | - | - | 0 |  |
|  |  | I5 | 1 | - | - | - | 1 (0) | 0 |  |
|  |  | I6 | 1 | - | - | - | 1 (0) | 0 |  |
|  |  | I7 | 1 | - | - | - | 1 (0) | 0 |  |
|  |  | I8 | 1 | - | - | - | 1 (0) | 0 |  |
|  |  | I9 | 1 | - | - | - | 1 (0) | 0 |  |
|  |  | I10 | 1 | - | - | - | 1 (0) | 0 |  |
|  |  | I11 | 1 | 1 (0) | - | - | - | 0 |  |
|  |  | I12 | 1 | 1 (1) | - | - | - | 1 |  |
|  |  | I13 | 1 | - | - | - | 1 (0) | 0 |  |
|  |  | I14 | 1 | - | - | - | 1 (0) | 0 |  |
|  |  | I15 | 1 | 1 (1) | - | - | - | 1 |  |
|  |  | I16 | 1 | 1 (0) | - | - | - | 0 |  |
|  |  | I17 | ND | - | 1 (0) | - | - | 0 |  |
|  |  | I18 | 1 | 1(1) | - | - | - | 1 |  |
| **Podlasie (J)** | 28 | J1 | ND | - | 1 (1) | - | - | ND | 13 (46.43) |
|  |  | J2 | 187 | 96 (12) | 1 (1) | 187 (158) | - | 158 |  |
|  |  | J3 | ND | - | 1 (1) | 2 (0) | - | ND |  |
|  |  | J4 | 23 | 23 (1) | - | - | - | 1 |  |
|  |  | J5 | ND | 1 (0) | 1 (0) | - | - | 0 |  |
|  |  | J6 | ND | - | 1 (1) | 3 (0) |  | ND |  |
|  |  | J7 | 1 | 1 (0) | - | - | - | 0 |  |
|  |  | J8 | 3 | 3 (1) | - | - | - | 1 |  |
|  |  | J9 | 1 | 1 (0) | - | - | - | 0 |  |
|  |  | J10 | 1 | 1 (0) | - | - | - | 0 |  |
|  |  | J11 | 1 | 1 (1) | - | - | - | 1 |  |
|  |  | J12 | 1 | 1 (0) | - | - | - | 0 |  |
|  |  | J13 | 1 | 1 (0) | - | - | - | 0 |  |
|  |  | J14 | 1 | 1 (0) | - | - | - | 0 |  |
|  |  | J15 | ND | - | 1 (1) | - | - | ND |  |
|  |  | J16 | ND | - | 1 (0) | - | - | 0 |  |
|  |  | J17 | 1 | 1 (0) | - | - | - | 0 |  |
|  |  | J18 | 1 | 1 (0) | - | - | - | 0 |  |
|  |  | J19 | 1 | 1 (1) | - | - | - | 1 |  |
|  |  | J20 | 1 | 1 (1) | - | - | - | 1 |  |
|  |  | J21 | ND | - | 1 (0) | - | - | 0 |  |
|  |  | J22 | ND | 15 (4) | 1 (1) | - | - | 4 |  |
|  |  | J23 | 1 | 1 (1) | - | - | - | 1 |  |
|  |  | J24 | 1 | 1 (0) | - | 1 (0) | - | 0 |  |
|  |  | J25 | 1 | 1 (0) | - | - | - | 0 |  |
|  |  | J26 | ND | - | 1 (0) | - | - | 0 |  |
|  |  | J27 | 1 | 1 (0) | - | - | - | 0 |  |
|  |  | J28 | ND | - | 1 (1) | - | - | ND |  |
| **Pomerania (K)** | 14 | K1 | 1 | 1 (0) | - | - | - | 0 | 2 (14.29) |
|  |  | K2 | 1 | 1(0) | - | - | - | 0 |  |
|  |  | K3 | 1 | 1 (0) | - | - | - | 0 |  |
|  |  | K4 | 1 | 1 (1) | - | - | - | 1 |  |
|  |  | K5 | 1 | 1 (0) | - | - | - | 0 |  |
|  |  | K6 | 1 | 1 (0) | - | - | - | 0 |  |
|  |  | K7 | 3 | 3 (0) | - | - | - | 0 |  |
|  |  | K8 | 1 | 1 (0) | - | - | - | 0 |  |
|  |  | K9 | ND | - | 1 (0) | - | - | 0 |  |
|  |  | K10 | ND | - | 1 (0) | - | - | 0 |  |
|  |  | K11 | 1 | 1 (1) | - | - | - | 1 |  |
|  |  | K12 | ND | - | 1 (0) | - | - | 0 |  |
|  |  | K13 | 1 | 1 (0) | - | - | - | 0 |  |
|  |  | K14 | ND | - | 1 (0) | - | - | 0 |  |
| **Świętokrzyskie (L)** | 6 | L1 | 3 | 3 (0) | - | - | - | 0 | 2 (33.33) |
|  |  | L2 | 1 | - | - | 1 (0) | - | 0 |  |
|  |  | L3 | ND | - | 1 (0) | - | - | 0 |  |
|  |  | L4 | ND | - | 1 (1) | - | - | ND |  |
|  |  | L5 | 50 | 38 (8) | 1 (1) | 12 (0) | - | 8 |  |
|  |  | L6 | 1 | 1 (0) | - | - | - | 0 |  |
| **Warmia-Masuria (M)** | 17 | M1 | 1 | 1 (1) | - | 1 (1) | - | 1 | 9 (52.94) |
|  |  | M2 | ND | - | 1 (0) | - | - | 0 |  |
|  |  | M3 | 1 | - | - | - | 1 (0) | 0 |  |
|  |  | M4 | ND | - | 1 (1) | - | - | ND |  |
|  |  | M5 | 2 | 2 (0) | - | - | - | 0 |  |
|  |  | M6 | 7 | 7 (0) | - | - | - | 0 |  |
|  |  | M7 | 1 | - | - | 1 (0) | - | 0 |  |
|  |  | M8 | 68 | 68 (10) | - | 68 (0) | - | 10 |  |
|  |  | M9 | 7 | 3 (0) | - | 4 (0) | - | 0 |  |
|  |  | M10 | 1 | 1 (0) | - |  | - | 0 |  |
|  |  | M11 | ND | - | 1 (1) | - | - | ND |  |
|  |  | M12 | ND | - | 1 (1) | - | - | ND |  |
|  |  | M13 | 1 | 1 (0) | - | - | - | 0 |  |
|  |  | M14 | 1 | 1 (1) | - | - | - | 1 |  |
|  |  | M15 | 3 | 3 (1) | - | - | - | 1 |  |
|  |  | M16 | ND | - | 1 (1) | - | - | ND |  |
|  |  | M17 | ND | - | 1 (1) | - | - | ND |  |
| **Greater Poland (N)** | 13 | N1 | 3 | 3 (2) | - | - | - | 2 | 5 (38.46) |
|  |  | N2 | 1 | 1 (0) | - | - | - | 0 |  |
|  |  | N3 | 4 | 4 (1) | - | - | - | 1 |  |
|  |  | N4 | 1 | 1 (0) | - | - | - | 0 |  |
|  |  | N5 | 1 | 1 (0) | - | - | - | 0 |  |
|  |  | N6 | 1 | 1 (1) | - | - | - | 1 |  |
|  |  | N7 | 1 | 1 (0) | - | - | - | 0 |  |
|  |  | N8 | 1 | 1 (0) | - | - | - | 0 |  |
|  |  | N9 | 1 | 1 (0) | - | - | - | 0 |  |
|  |  | N10 | 111 | 111 (34) | 1 (1) | - | - | 34 |  |
|  |  | N11 | ND | - | 1 (0) | - | - | 0 |  |
|  |  | N12 | ND | - | 1 (0) | - | - | 0 |  |
|  |  | N13 | 1 | 1 (1) | - | - | - | 1 |  |
| **West Pomerania (O)** | 3 | O1 | ND | 4 (1) | 1 (1) | 4 (0) | 5 (0) | ND | 3 (100) |
|  |  | O2 | ND | - | 1 (1) | - | - | ND |  |
|  |  | O3 | ND | - | 1 (1) | - | - | ND |  |
| **SUMA** | **279** | **-** | **-** | **897 (165)** | **101 (40)** | **409 (166)** | **32 (5)** | **-** | **88** |
