# Supplementary material for: Shedding and genetic diversity of Coxiella burnetii in Polish dairy cattle
Source: PLoS One. 2019 Jan 10;14(1):e0210244. doi: 10.1371/journal.pone.0210244 (PMC6328121; doi:10.1371/journal.pone.0210244)
Supplement: S2 Table — (DOCX) [file pone.0210244.s002.docx]

**S2 Table.** Type of tested milk and dairy products and qPCR results.

| **Type of dairy product** | **Manufacturer’s ID** | **Ct values -**  **real-time PCR** |
| --- | --- | --- |
| **Milk** | | |
| raw milk (from vending machine) | ML1 | 30.42 |
|  | ML2 | negative |
|  | ML3 | negative |
|  | ML4 | negative |
|  | ML5 | negative |
|  | ML6 | negative |
|  | ML7 | negative |
|  | ML8 | negative |
|  | ML9 | negative |
|  | ML10 | negative |
| whole milk (3,2%) | PR1 | negative |
|  | PR3 | 33.01 |
|  | PR4 | 33.91 |
|  | PR5 | 35.2 |
|  | PR6 | 34.98 |
|  | PR7 | 32 |
|  | PR8 | 34.36 |
|  | PR34 | 35.55 |
|  | PR38 | negative |
|  | PR40 | 33.81 |
| low-fat milk (2%) | PR1 | negative |
|  | PR5 | 35.4 |
|  | PR6 | 35.02 |
|  | PR8 | 34.37 |
|  | PR10 | 34.38 |
|  | PR11 | 34.08 |
|  | PR30 | negative |
|  | PR34 | 34.72 |
|  | PR40 | 35.16 |
|  | PR38 | 33.01 |
| **Milk beverages** | | |
| flavored milk | PR2 | 34.34 |
|  | PR5 | negative |
| yogurt | PR2 | 34.58 |
|  | PR3 | 33.17 |
|  | PR5 | 33.29 |
|  | PR6 | 32.98 |
|  | PR8 | 35.12 |
|  | PR9 | 32.58 |
|  | PR10 | 33.46 |
|  | PR11 | negative |
|  | PR12 | 33.8 |
|  | PR13 | 35.77 |
|  | PR14 | 35.86 |
|  | PR15 | 35.47 |
|  | PR16 | negative |
|  | PF30 | 34.64 |
|  | PR33 | 33.96 |
|  | PR34 | negative |
|  | PR37 | 32.89 |
|  | PR38 | 34.05 |
|  | PR40 | 33.18 |
|  | PR43 | negative |
| kefir | PR3 | negative |
|  | PR5 | 34.42 |
|  | PR6 | 34.18 |
|  | PR33 | negative |
| buttermilk | PR12 | 33.78 |
|  | PR13 | 34.33 |
| sour milk | PR6 | negative |
|  | PR33 | negative |
| **Creams** | | |
| low-fat sour cream (12%) | PR17 | 35.99 |
| low-fat sour cream (12%) | PR33 | negative |
| fat sour cream (18%) | PR34 | negative |
| fat sour cream (18%) | PR35 | negative |
| fat sour cream (18%) | PR36 | 32.45 |
| fat sour cream (18%) | PR30 | negative |
| whipping cream 30% | PR11 | negative |
| **Fresh cheeses** | | |
| cheese curds | PR21 | 33.86 |
| cheese curds | PR29 | 33.31 |
| cottage cheese | PR18 | 33.22 |
| cottage cheese | PR30 | 33.27 |
| cottage cheese | PR38 | 29.98 |
| cream cheese | PR18 | 33.36 |
| cream cheese | PR32 | 30.79 |
| farmer’s cheese | PR32 | 31.02 |
| feta cheese | PR34 | 34.9 |
| cream cheese | PR19 | 29.43 |
| cream cheese | PR39 | 31.14 |
| cream cheese | PR43 | negative |
| cream cheese | PR46 | 35.3 |
| Italian type cheese | PR27 | 35.78 |
| mascarpone | PR40 | 32.09 |
| mozzarella | PR18 | 35.18 |
| processed cheese with mozzarella | PR23 | 34.42 |
| quark cheese | PR17 | negative |
| ricotta | PR34 | 33.85 |
| smoked cheese | PR21 | 30.58 |
| **Soft-ripened cheeses and hard-ripened cheeses** | | |
| cheese1 | PR5 | 31.18 |
| cheese2 | PR20 | 34.38 |
| cheese3 | PR22 | 32.71 |
| cheese4 | PR25 | 33.09 |
| cheese5 | PR26 | 30.81 |
| cheese6 | PR34 | 30.28 |
| cheese7 | PR39 | 33.11 |
| cheese8 | PR45 | 31.14 |
| cheese9 | PR18 | 31.43 |
| cheese10 | PR42 | 34.54 |
| cheese11 | PR44 | 32.38 |
| cheese12 | PR47 | 32.71 |
| cheese13 | PR29 | 33.63 |
| cheese14 | PR27 | 32.18 |
| blue-veined cheese | PR24 | negative |
| blue-veined cheese | PR41 | 30.2 |
| brie cheese | PR28 | negative |
| brie cheese | PR47 | negative |
| camembert cheese | PR31 | 30.79 |
| camembert cheese | PR34 | negative |
