# Supplementary material for: Shedding and genetic diversity of Coxiella burnetii in Polish dairy cattle
Source: PLoS One. 2019 Jan 10;14(1):e0210244. doi: 10.1371/journal.pone.0210244 (PMC6328121; doi:10.1371/journal.pone.0210244)
Supplement: S3 Table — ND ‒ not determined, due to lack of product amplification for all tested loci *according to nomenclature proposed by Tilburg [35] (DOCX) [file pone.0210244.s003.docx]

**S3 Table.** Results of MLVA genotyping.

| **No.** | **Herd ID** | **Type of sample** | **Ct**  **real-time PCR** | **No. of STR’s in locus** | | | | | | **Genotype of MLVA*** |
| --- | --- | --- | --- | --- | --- | --- | --- | --- | --- | --- |
|  |  |  |  | **Ms**  **23** | **Ms 24** | **Ms**  **27** | **Ms**  **28** | **Ms**  **33** | **Ms**  **34** |  |
|  | B1 | BTM | 30.97 | 6 | 13 | 2 | 7 | - | 9 | ND |
|  | B10 | BTM | 32.13 | - | 14 | - | 7 | - | - | ND |
|  | B11 | BTM | 31.28 | 6 | 13 | 2 | 7 | 9 | 10 | J |
|  | B12 | individual milk | 31.14 | 6 | 13 | - | 7 | 9 | 8 | ND |
|  | B15 | BTM | 29.3 | 6 | 13 | 2 | 7 | 9 | 8/10 | mixed |
|  | B20 | placenta | 14.11 | 6 | 13 | 2 | 7 | 9 | 9 | I |
|  | B23 | individual milk | 30.88 | 6 | 13 | 2 | 7 | 9 | 9 | I |
|  | B28 | individual milk | 33.74 | - | - | 2 | 7 | 9 | - | ND |
|  | B36 | BTM | 27.16 | 6 | 13 | 2 | 7 | 9 | 10 | J |
|  | B39 | individual milk | 24.01 | 6 | 13 | 2 | 7 | 9 | 9 | I |
|  | C12 | individual milk | 27.56 | 6 | 13 | 2 | 7 | 9 | 10 | J |
|  | C16 | BTM | 31.33 | 6 | 13 | - | - | 9 | 8 | ND |
|  | C17 | individual milk | 30.2 | 6 | 13 | 2 | 7 | 9 | 8 | BG |
|  | C21 | BTM | 32.26 | - | 13 | - | 7 | - | 9 | ND |
|  | C23 | individual milk | 32.41 | - | 13 | - | 7 | - | 9 | ND |
|  | C24 | individual milk | 30.29 | 6 | 13 | 2 | 7 | 9 | 10 | J |
|  | C44 | individual milk | 23.66 | 6 | 13 | 2 | 7 | 9 | 9 | I |
|  | **C55** | BTM | **28.89** | **6** | **14** | **2** | **7** | **9** | **9** | **PL1** |
|  | D3 | BTM | 27.73 | 6 | 12 | 2 | 7 | 9 | 9 | BE |
|  | E12 | BTM | 29.82 | 9 | 27 | 4 | 6 | 9 | 5 | NM |
|  | E13 | individual milk | 31.94 | 6 | 13 | - | 7 | 9 | 11 | ND |
|  | G13 | BTM | 31.51 | 6 | - | - | - | - | 8 | ND |
|  | G14 | individual milk | 30 | 6 | 13 | 2 | 7 | 9 | 10 | J |
|  | G20 | individual milk | 31.81 | 6 | 13 | 2 | 7 | 9 | 10 | J |
|  | G22 | individual milk | 27.38 | 6 | 13 | 2 | 7 | 9 | 9 | I |
|  | H3 | individual milk | 27.63 | 6 | 13 | 2 | 7 | 9 | 9 | I |
|  | H6 | individual milk | 26.26 | 6 | 13 | 2 | 7 | 9 | 8 | BG |
|  | H7 | BTM | 28.33 | 6 | 13 | 2 | 7 | 9 | 8 | BG |
|  | H8 | individual milk | 23.71 | 6 | 13 | 2 | 7 | 9 | 9 | I |
|  | J2 | individual milk | 26.37 | 6 | 13 | 2 | 7 | 9 | 8 | BG |
|  | J3 | BTM | 32.92 | - | 13 | - | - | - | - | ND |
|  | J4 | individual milk | 27.19 | 6 | 13 | 2 | 7 | 9 | 10 | J |
|  | J11 | individual milk | 30.07 | - | - | 2 | 7 | - | - | ND |
|  | J20 | individual milk | 32.33 | - | 14 | 2 | - | 9 | 9 | ND |
|  | J22 | individual milk | 26.96 | 6 | 13 | 2 | 7 | 9 | 9 | I |
|  | J28 | BTM | 32.29 | - | 13 | - | - | 9 | - | ND |
|  | L5 | individual milk | 26.87 | 6 | 13 | 2 | 7 | 9 | 8 | BG |
|  | M1 | individual milk | 27.41 | 6 | 13 | 2 | 7 | 9 | 9 | I |
|  | M4 | BTM | 31.57 | 6 | 13 | 2 | 7 | 9 | 8 | BG |
|  | M8 | individual milk | 23.19 | 6 | 13 | 2 | 7 | 9 | 9 | I |
|  | M11 | BTM | 30.52 | 6 | 13 | - | 7 | - | 8/9 | mixed |
|  | M14 | individual milk | 30.46 | 6 | 13 | 2 | 7 | 9 | 9 | I |
|  | M15 | individual milk | 29.43 | 6 | 13 | 2 | 7 | 9 | 9 | I |
|  | M16 | BTM | 31.74 | - | 12 | - | 7 | - | 11 | ND |
|  | M17 | BTM | 30.38 | 6 | 13 | 2 | - | 9 | 8 | ND |
|  | **N3** | individual milk | **26.81** | **6** | **13** | **2** | **7** | **9** | **7** | **PL2** |
|  | N10 | individual milk | 26.95 | 6 | 12 | 2 | 7 | 9 | 9 | BE |
|  | O1 | individual milk | 29.38 | 6 | 13 | 2 | 7 | 9 | 10 | J |
|  | O2 | BTM | 32.92 | - | 14 | 2 | 7 | - | - | ND |
