# Supplementary material for: Shedding and genetic diversity of Coxiella burnetii in Polish dairy cattle
Source: PLoS One. 2019 Jan 10;14(1):e0210244. doi: 10.1371/journal.pone.0210244 (PMC6328121; doi:10.1371/journal.pone.0210244)
Supplement: S4 Table — ND ‒ not determined, due to lack of product amplification for all tested loci *according to nomenclature proposed by Tilburg [35] (DOCX) [file pone.0210244.s004.docx]

**S4 Table.** Results of MLVA genotyping of milk and dairy products.

| **No.** | **Type of dairy products** | **Manufacture’s ID** | **Ct**  **real-time PCR** | **No. of STRs in locus** | | | | | | **Genotype of MLVA*** |
| --- | --- | --- | --- | --- | --- | --- | --- | --- | --- | --- |
|  |  |  |  | **Ms**  **23** | **Ms**  **24** | **Ms**  **27** | **Ms**  **28** | **Ms**  **33** | **Ms**  **34** |  |
| 1. | raw milk | ML1 | 30.42 | 6 | 13 | 2 | 7 | 9 | 9 | I |
| 2. | hard-ripened cheese | PR5 | 31.18 | 6 | 13/14 | - | 7 | 9 | 11/12 | mixed |
| 3. | pasterised milk | PR7 | 32 | 6 | 13 | - | - | 9 | - | ND |
| 4. | yogurt | PR9 | 32.58 | 6 | 13 | 2 | 7 | - | 9 | ND |
| 5. | cream cheese | PR19 | 29.43 | 6 | 13 | 2 | 7 | 9 | 9 | I |
| 6. | smoked cheese | PR21 | 30.58 | - | 13 | - | 7 | - | 8/9/10 | mixed |
| 7. | hard-ripened cheese | PR26 | 30.81 | 6 | 12/13 | 2 | 7 | 9 | 9/11 | mixed |
| 8. | camembert cheese | PR31 | 31.02 | **6** | **12** | **2** | **7** | **9** | **12** | **PL3** |
