# Supplementary material for: Shedding and genetic diversity of Coxiella burnetii in Polish dairy cattle
Source: PLoS One. 2019 Jan 10;14(1):e0210244. doi: 10.1371/journal.pone.0210244 (PMC6328121; doi:10.1371/journal.pone.0210244)
Supplement: S5 Table — ND–not determined *Sequence type established based on incomplete allelic profile ** according to online database [26] (DOCX) [file pone.0210244.s005.docx]

**S5 Table.** Results of genotyping using MST method.

| **No.** | **Type of dairy products** | **Manufacture’s ID** | **Ct**  **real-time PCR** | **Alleles identified in spacers**** | | | | | | | | | | **Sequence type** |
| --- | --- | --- | --- | --- | --- | --- | --- | --- | --- | --- | --- | --- | --- | --- |
|  |  |  |  | **Cox** **2** | **Cox 5** | **Cox 18** | **Cox** **20** | **Cox 22** | **Cox 37** | **Cox** **51** | **Cox** **56** | **Cox 57** | **Cox 61** |  |
|  | B1 | BTM | 30.97 | 3 | 2 | 6 | 1 | 5 | **10** | 4 | 10 | - | 5 | ST61* |
|  | B10 | BTM | 32.13 | 3 | - | - | - | - | - | - | - | - | - | ND |
|  | B11 | BTM | 31.28 | 3 | 2 | 6 | - | 5 | **10** | 4 | 10 | - | - | ST61* |
|  | B12 | individual milk | 31.14 | 3 | 2 | 6 | 1 | 5 | **10** | 4 | 10 | - | 5 | ST61* |
|  | B15 | BTM | 29.3 | 3 | 2 | 6 | 1 | 5 | **10** | 4 | 10 | 6 | 5 | ST61 |
|  | B20 | placenta | 14.11 | 3 | 2 | 6 | 1 | 5 | **10** | 4 | 10 | 6 | 5 | ST61 |
|  | B23 | individual milk | 30.88 | 3 | 2 | 6 | 1 | 5 | **10** | 4 | 10 | 6 | 5 | ST61 |
|  | B28 | individual milk | 33.74 | 3 | - | - | - | 5 | - | - | - | - | 5 | ND |
|  | B36 | BTM | 27.16 | 3 | 2 | 6 | 1 | 5 | **10** | 4 | - | - | 5 | ST61* |
|  | B39 | individual milk | 24.01 | 3 | 2 | 6 | 1 | 5 | **10** | 4 | 10 | 6 | 5 | ST61 |
|  | C12 | individual milk | 27.56 | 3 | 2 | 6 | 1 | 5 | **10** | 4 | 10 | 6 | 5 | ST61 |
|  | C16 | BTM | 31.33 | 3 | - | 6 | - | 5 | **10** | 4 | - | 6 | - | ST61* |
|  | C17 | individual milk | 30.2 | 3 | 2 | 6 | - | 5 | - | 4 | 10 | - | - | ST20/ST61* |
|  | C21 | BTM | 32.26 | 3 | - | - | - | - | **10** | - | - | - | 5 | ND |
|  | C23 | individual milk | 32.41 | 3 | 2 | 6 | - | 5 | **10** | 4 | 10 | - | 5 | ST61* |
|  | C24 | individual milk | 30.29 | 3 | 2 | - | - | 5 | **10** | 4 | 10 | - | 5 | ST61* |
|  | C44 | individual milk | 23.66 | 3 | 2 | 6 | 1 | - | **10** | 4 | 10 | - | 5 | ST61* |
|  | C55 | BTM | 28.89 | 3 | 2 | 6 | 1 | 5 | **10** | 4 | 10 | 6 | 5 | ST61 |
|  | D3 | BTM | 27.72 | 3 | 2 | 6 | 1 | 5 | **10** | 4 | 10 | 6 | 5 | ST61 |
|  | E12 | BTM | 29.82 | 3 | 8 | 5 | 3 | 4 | 1 | 6 | 7 | 6 | 5 | ST16 |
|  | E13 | individual milk | 31.94 | 3 | 2 | 6 | 1 | 5 | **10** | 4 | - | - | 5 | ST61* |
|  | G13 | BTM | 31.51 | - | 2 | - | - | - | - | - | - | - | - | ND |
|  | G14 | individual milk | 30 | 3 | 2 | 6 | 1 | 5 | **10** | 4 | 10 | 6 | 5 | ST61 |
|  | G20 | individual milk | 31.81 | 3 | 2 | 6 | 1 | 5 | **10** | 4 | 10 | 6 | 5 | ST61 |
|  | G22 | individual milk | 27.38 | 3 | 2 | 6 | 1 | 5 | **10** | 4 | - | - | 5 | ST61* |
|  | H3 | individual milk | 27.63 | 3 | 2 | 6 | 1 | 5 | **10** | 4 | 10 | 6 | 5 | ST61 |
|  | H6 | individual milk | 26.6 | - | 2 | 6 | - | 5 | **10** | 4 | 10 | - | 5 | ST61* |
|  | H7 | BTM | 28.33 | - | 2 | 6 | 1 | 5 | **10** | 4 | 10 | - | 5 | ST61* |
|  | H8 | individual milk | 23.71 | 3 | 2 | 6 | 1 | 5 | **10** | 4 | 10 | 6 | 5 | ST61 |
|  | J2 | individual milk | 26.37 | 3 | 2 | 6 | 1 | 5 | **10** | 4 | 10 | 6 | 5 | ST61 |
|  | J3 | BTM | 32.92 | **-** | 2 | **-** | **-** | 5 | **-** | **-** | **-** |  | **-** | ND |
|  | J4 | individual milk | 27.19 | 3 | 2 | 6 | 1 | 5 | **10** | 4 | 10 | - | 5 | ST61* |
|  | J11 | individual milk | 30.07 | **-** | **-** | **-** | **-** | **-** | **10** | **-** | **-** | **-** | **-** | ND |
|  | J20 | individual milk | 32.33 | 3 | 2 | 6 | 1 | 5 | **10** | - | 10 | - | 5 | ST61* |
|  | J22 | individual milk | 26.96 | 3 | 2 | 6 | - | 5 | **10** | 4 | 10 | - | 5 | ST61* |
|  | J28 | BTM | 32.39 | 3 | - | - | 1 | 5 | - | - | - | - | 5 | ND |
|  | L5 | individual milk | 26.87 | 3 | 2 | 6 | 1 | 5 | **10** | 4 | 10 | 6 | 5 | ST61 |
|  | M1 | individual milk | 27.41 | 3 | 2 | 6 | 1 | 5 | **10** | 4 | 10 | - | 5 | ST61* |
|  | M4 | BTM | 31.57 | 3 | 2 | 6 | 1 | 5 | **10** | 4 | 10 | - | 5 | ST61* |
|  | M8 | individual milk | 23.26 | 3 | 2 | 6 | 1 | 5 | **10** | 4 | 10 | 6 | 5 | ST61 |
|  | M11 | BTM | 30.52 | 3 | 2 | 6 | - | 5 | **10** | 4 | - | - | - | ST61* |
|  | M14 | individual milk | 30.46 | 3 | - | 6 | - | 5 | **10** | 4 | 10 | - | 5 | ST61* |
|  | M15 | individual milk | 29.46 | 3 | 2 | 6 | 1 | 5 | **10** | 4 | 10 | 6 | 5 | ST61 |
|  | M16 | BTM | 31.74 | 3 | 2 | 6 | 1 | 5 | - | - | 10 | - | 5 | ST20/ST61* |
|  | M17 | BTM | 30.38 | 3 | 2 | 6 | 1 | 5 | **10** | 4 | 10 | - | 5 | ST61* |
|  | N3 | individual milk | 26.81 | 3 | 2 | 6 | 1 | 5 | **10** | 4 | 10 | 6 | 5 | ST61 |
|  | N10 | individual milk | 26.95 | 3 | 2 | 6 | 1 | 5 | **10** | 4 | 10 | 6 | 5 | ST61 |
|  | O1 | individual milk | 29.38 | 3 | 2 | 6 | 1 | 5 | **10** | 4 | 10 | - | 5 | ST61* |
|  | O2 | BTM | 32.92 | **-** | 2 | **-** | **-** | **-** | **-** | **-** | **-** |  | **-** | ND |
