# Supplementary material for: Shedding and genetic diversity of Coxiella burnetii in Polish dairy cattle
Source: PLoS One. 2019 Jan 10;14(1):e0210244. doi: 10.1371/journal.pone.0210244 (PMC6328121; doi:10.1371/journal.pone.0210244)
Supplement: S6 Table — ND–not determined *Sequence type established based on incomplete allelic profile ** according to online database [26] (DOCX) [file pone.0210244.s006.docx]

**S6 Table.** Results of genotyping of milk and dairy products using MST method.

| **No.** | **Type of dairy products** | **Manufacture’s ID** | **Ct**  **real-time PCR** | **Alleles identified in spacers**** | | | | | | | | | | **Sequence type** |
| --- | --- | --- | --- | --- | --- | --- | --- | --- | --- | --- | --- | --- | --- | --- |
|  |  |  |  | **Cox** **2** | **Cox 5** | **Cox 18** | **Cox** **20** | **Cox 22** | **Cox 37** | **Cox** **51** | **Cox** **56** | **Cox 57** | **Cox 61** |  |
| 1 | raw milk | M1 | 30.42 | 3 | 2 | 6 | 1 | - | **10** | 4 | 10 | - | 5 | ST61* |
| 2 | hard-ripened cheese | PR5 | 31.18 | 3 | 2 | 6 | 1 | 5 | **10** | 4 | 10 | - | 5 | ST61* |
| 3 | pasterised milk | PR7 | 32 | 3 | - | - | - | 5 | **10** | 4 | - | - | - | ND |
| 4 | yogurt | PR9 | 32.58 | 3 | - | - | - | - | - | - | - | - | 5 | ND |
| 5 | cream cheese | PR19 | 29.43 | 3 | 2 | 6 | 1 | 5 | **10** | 4 | 10 | 6 | 5 | ST61 |
| 6 | smoked cheese | PR21 | 30.58 | 3 | - | 6 | 1 | 5 | **10** | 4 | 10 | - | - | ST61* |
| 7 | hard-ripened cheese | PR26 | 30.81 | 3 | - | - | 1 | - | **10** | 4 | 10 | - | 5 | ND |
| 8 | camembert cheese | PR31 | 30.79 | - | - | 6 | 1 | - | - | 4 | - | - | 5 | ND |
